# RiboSubstrates: a web application addressing the cleavage specificities of ribozymes in designated genomes

**DOI:** 10.1186/1471-2105-7-480

**Published:** 2006-10-31

**Authors:** Jean-François Lucier, Lucien Junior Bergeron, Francis P Brière, Rodney Ouellette, Sherif Abou Elela, Jean-Pierre Perreault

**Affiliations:** 1 RNA Group/Groupe ARN, Sherbrooke, Canada; 2 Département de microbiologie et infectiologie, Université de Sherbrooke, Sherbrooke, Canada; 3 Département de biochimie, Université de Sherbrooke, Sherbrooke, Canada; 4 Institut Atlantique de Recherche sur le Cancer, Moncton, Canada; 5 Département de Chimie et Biochimie, Université de Moncton, Moncton, Canada

## Abstract

**Background:**

RNA-dependent gene silencing is becoming a routine tool used in laboratories worldwide. One of the important remaining hurdles in the selection of the target sequence, if not the most important one, is the designing of tools that have minimal off-target effects (i.e. cleaves only the desired sequence). Increasingly, in the current dawn of the post-genomic era, there is a heavy reliance on tools that are suitable for high-throughput functional genomics, consequently more and more bioinformatic software is becoming available. However, to date none have been designed to satisfy the ever-increasing need for the accurate selection of targets for a specific silencing reagent.

**Results:**

In order to overcome this hurdle we have developed RiboSubstrates . This integrated bioinformatic software permits the searching of a cDNA database for all potential substrates for a given ribozyme. This includes the mRNAs that perfectly match the specific requirements of a given ribozyme, as well those including Wobble base pairs and mismatches. The results generated allow rapid selection of sequences suitable as targets for RNA degradation. The current web-based RiboSubstrates version permits the identification of potential gene targets for both SOFA-HDV ribozymes and for hammerhead ribozymes. Moreover, a minimal template for the search of siRNAs is also available. This flexible and reliable tool is easily adaptable for use with any RNA tool (i.e. other ribozymes, deoxyribozymes and antisense), and may use the information present in any cDNA bank.

**Conclusion:**

RiboSubstrates should become an essential step for all, even including "non-RNA biologists", who endeavor to develop a gene-inactivation system.

## Background

The development of gene-inactivation systems is an active and important field for both functional genomics and gene therapy (reviewed in references [1-3]). In fact, RNA dependent gene silencing is becoming a routine tool used by laboratories worldwide. However, the issue of substrate specificity remains an important hurdle, regardless of the approach used (e.g. see references [4-7]). A key question is how to design target-specific RNA molecules that do not produce any side effects. There are several potential sources for side effects, including triggerring immunological responses, as well as degradation and translational suppression, to name only these ones. Moreover, in many cases side effects may result from the cleavage of non-desired mRNA species. There are several means for verifying the specificity of a given RNA, including profiling its impact on global gene expression using microarray analysis [4,6,7]. However, all of these approaches are performed following the silencing experiments and, consequently, have no predictive value. Only in the case of siRNA, have algorithms been developed to identify potential targeting of non-desired mRNA species before to attempting any manipulation [e.g. [8,9]]. However, since it is uncertain how siRNAs cause off-target effects, the accuracy of these algorithms remains to be demonstrated. Moreover, no such algorithm has been reported for ribozyme design. Thus, the selection of a unique target sequence remains a key process that, in most cases, is determined empirically.

Ideally, we would like to possess either a bioinformatic or a biochemical approach that permits verification of whether or not an RNA silencing tool offers the specificity required in order to target a unique mRNA species. This should permit the identification of all potential substrates, including both those that are perfectly matching as well those for which binding involves either Wobble base pairs or the presence of some mismatches. The commonly used Blast based target search is of limited utility in this regard because it is not suitable for evaluating short sequences like that found in the Hepatitis Delta Virus ribozyme (HDV ribozyme) binding domain (i.e. a stretch of 7 nucleotides) [7]. More specifically, the Blastn algorithm uses an heuristic approach to search for a minimal sequence size of 7 consecutive nucleotides which cannot contain any mismatches [10]. Alternatively, there is the WU-BLASTalgorithm that supports word-length search as small as only 1 nucleotide [11]. However, this algorithm is not suitable for RNA target search since it can miss potential hits. For example, if the searched sequence is composed of only G and U nucleotides, because of wobble base pairing, the searched signature will be composed of Y and R nucleotides. With such a signature, WU-BLAST cannot find any hits because no perfectly matched nucleotide can be retreived. Finally, there is the Smith and Waterman algorithm for searching minimal sequence; however it is known to be slow and memory consuming [12]. Most importantly there is no software that offers predefined templates for specific RNA silencing tools. At the dawn of post-genomic era, where there is a heavy reliance on tools that are suitable for high-throughput functional genomics, more and more bioinformatic software is becoming available, but, to date, none have been designed to satisfy the ever increasing need for the fast and accurate selection of targets for a specific silencing reagent.

Here we describe "RiboSubstrates", a new solution for the selection of RNA targets. RiboSubstrates is a portable software that can be adapted to any kind of RNA silencing requirements, and may be used to scan any predefined cDNA database. The main goal of the program is to evaluate whether or not a given ribozyme sequence has a suitable and unique target sequence within the transcriptome of a particular genome. The results obtained by the RiboSubstrates scan provides an overall evaluation of the potential for non-specific cleavages, and thus helps to identify the ribozyme that should have the least amount of non-specific cleavage for a given application *in vivo*. To our knowledge, to date no other software permitting the identification of non-specific targets has been reported for ribozyme development. The current version of RiboSubstrates is a web-based application that permits the identification of potential gene targets for SOFA-HDV ribozymes, hammerhead ribozymes and siRNAs.

## Implementation

RiboSubstrates has been written in Perl using the freely available CPAN modules CGI::Application, HTML::Template and Class::DBI [13,14]. In order to facilitate the use of the software, a help document was developed and is accessible at any moment at the top of the search pages.

**Figure 1:**
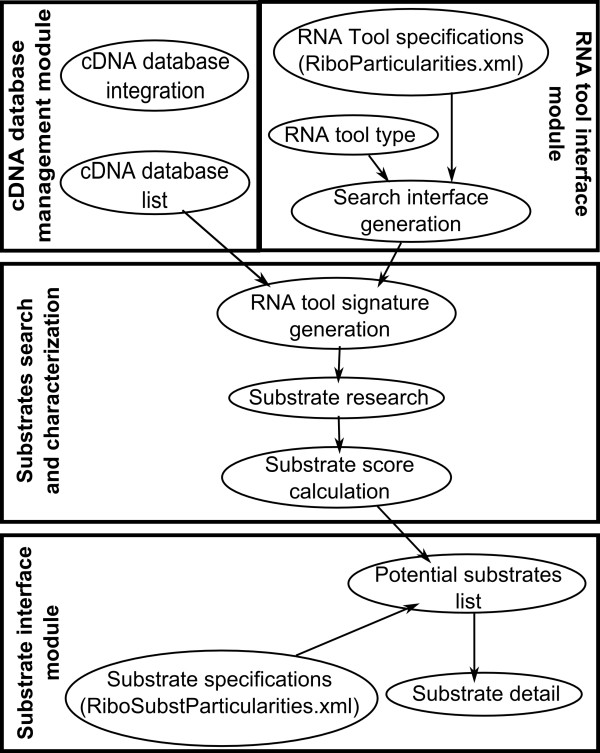
**Process flow for RiboSubstrates**. The cDNA database management module, the RNA tool interface module, the substrate search and characterization module and the substrate interface module are illustrated.

Essentially, RiboSubstrates includes four modules and two configuration files (see Figure 1). The first module was developed for new cDNA database integration. To date, the databases from *Homo sapiens*, *Mus musculus*, *Saccharomyces cerevisiae*, *Escherichia coli*, *Lactococcus lactis *and *Leishmania major *are available. Additional information on databases is available in the online help (see Database notes). These databases were recovered from public libraries and integrated into RiboSubstrates using the described module. In the case of *Lactococcus lactis *and *Leishmania major*, the mRNAs were extracted directly from the annotation of these genomes. Additional cDNA databases can easily be added on request; all that is required is a file in fasta format containing all of the entries. For a full RiboSubstrates integration, the header must be in the following format: >ID|Name|Description. The ID field must be the ID provided by the external source for a direct link to the external source description. The description can be anything; we used the gene function and location in genome for the default description. If the header is not in the above format, the search result will display the header without any interpretation.

The second module interprets a configuration file provided to the application (RiboParticularities.xml). This file describes the signature format to be searched for each ribozyme. For example, it will include information on the length of the recognition domain of a given ribozyme; the specific nucleotide requirement within, before, or after the binding domain, etc. Position constraints, including Watson-Crick and Wobble pairing, can be also considered. An illustration of the ribozymes, including the characteristics of the descriptors, is posted on the web page. A domain for a given ribozyme which a researcher is currently entering a sequence is simultaneously boxed in red in order to prevent any error. Researchers can enter sequences that include either Us or dTs without any difference.

The third module is the substrate search and score calculation. Initially, the cDNA database is scanned for the substrate sequence signature generated from the user inputs combined to the ribozyme constraints defined in the configuration file. Possible constraints include a maximum number of either Wobble base pairs or mismatches. In order to minimize the calculation time a maximum of 3 mismatches has been defined by default. This module produces a preliminary result file of potential substrates. A score is then calculated for each potential substrate. Each Wobble base pair encountered increases the score by 10, and each mismatch by 100. These arbitrarily values have been fixed based on kinetic data from different sources (e.g. see references [15,16] for the HDV ribozyme). For example, in the case of a SOFA-HDV ribozyme, the presence of a mismatch has been shown to reduce the overall specificity substrate from 5 to 18 times, while the presence of a Wobble base pair has been shown to have a limited effect. If the information for a spacer (see SOFA-HDV ribozyme example below) is specified in RiboParticularities.xml, then the tool score can be increased by the factors specified in the configuration file.

RiboSubstrates does not have a score cut off that indicates that a ribozyme should be re-designed because each RNA tool has its own specific requirements in order to be functional and the presence of both a Wobble base pair or a mismatch might affect different ribozymes differently. Moreover, the relationship between substrate specificity and RNA silencing efficacy is not defined by a simple linear equation based on the binding. The score serves mainly to help in identifying potential target sites including Wobble base pairs versus those possessing mismatches. Moreover, it is not realistic for a unique scoring system to be relevant for all potential RNA silencing tools since they all possess different requirements in terms of substrate specificity. However, regardless of the specific case, the presence of a mismatch will be more detrimental than the presence of a Wobble base pair.

**Figure 2:**
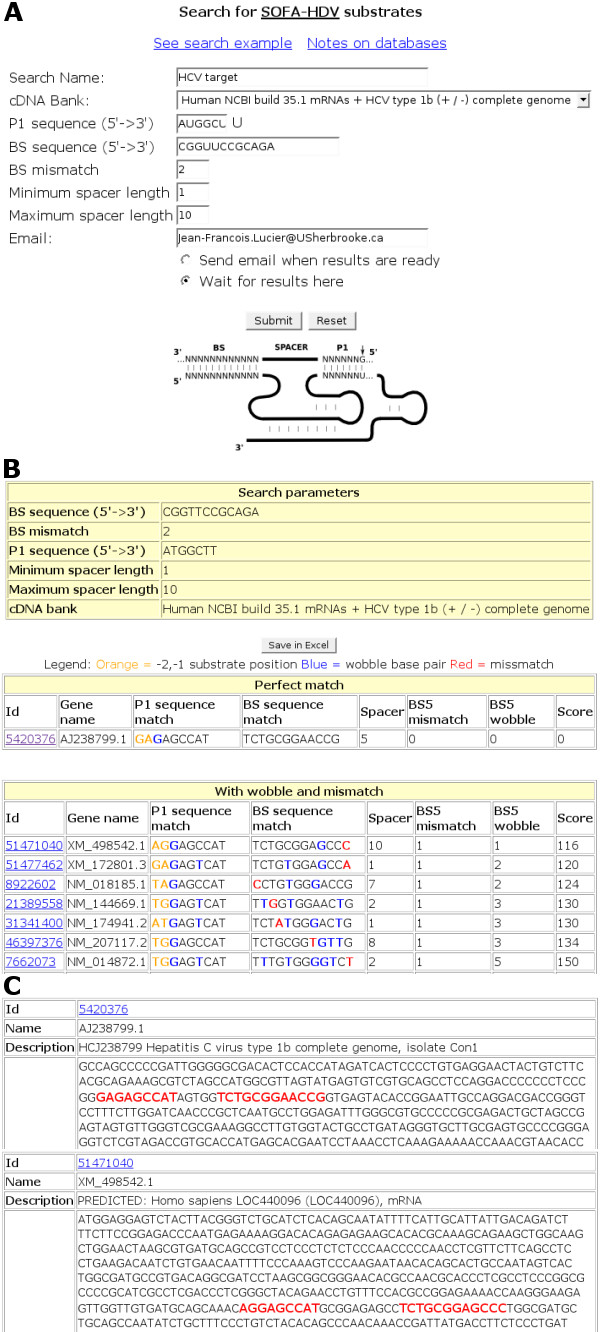
**Design of RiboSubstrates with the parameters for the SOFA-HDV ribozyme**. (**A**) Secondary structure and nucleotide sequence of the SOFA-HDV ribozyme targeting an mRNA. The P1 stem of the ribozyme and the biosensor (BS) of the SOFA module are identified. The letter N indicates the presence of an A, C, G or T residue. The arrow indicates the cleavage site. (**B**) The RiboSubstrates input with the signature to be searched for the SOFA-HDV-Rz targeting the HCV virus (SOFA-HDV-Rz-135) and the substrate display table. Mismatched nucleotides and Wobble base pairs are identified in red and blue, respectively. (**C**) The more detailed tables for both the mRNA encoding the HCV genome 1b and the predicted *Homo sapiens *LOC440096, respectively.

**Figure 3:**
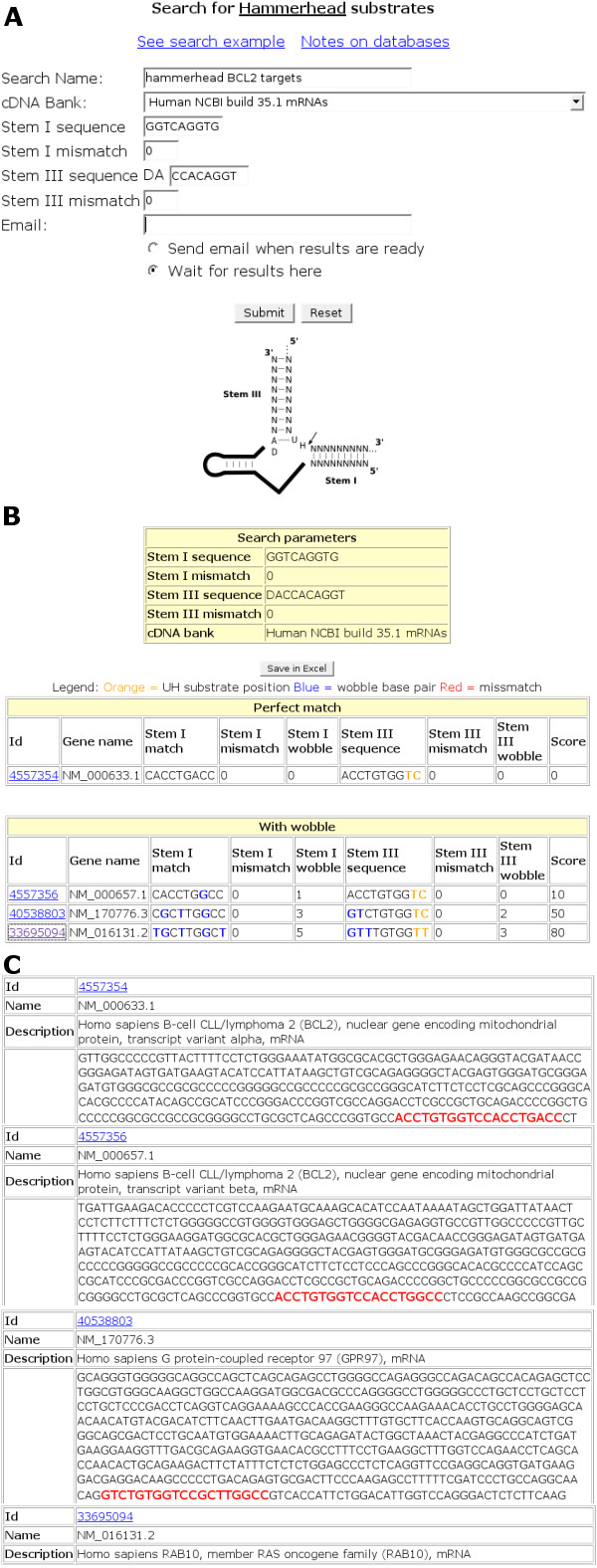
**Design of RiboSubstrates with the parameters for the hammerhead ribozyme**. (**A**) Secondary structure and nucleotide sequence of the hammerhead ribozyme targeting an mRNA. The two binding domains of the ribozyme are identified. The letter N indicates the presence of an A, C, G or T residue. The arrow indicates the cleavage site. (**B**) The RiboSubstrates input with the signature to be searched for the hammerhead ribozyme targeting the *Homo sapiens *Bcl-2 mRNA (hhRz-Bcl-2-1). Mismatched nucleotides and Wobble base pairs are identified in red and blue, respectively. (**C**) The substrate display table retrieved for this ribozyme.

Finally, the fourth module is the substrates list and detailed display. The list display is generated using a second configuration file (RiboSubstParticularities.xml) that describes the substrate list display. Each substrate sequence has different constraints for color mapping based on nucleotide binding to ribozyme. The color mapping is based on position, mismatches and wobble. Result tables are sorted as a function of the score, the lowest scores being the best matches. The first result table contains all substrates that are perfectly matched for the RNA tool in question (Figures 2 and 3). The second table contains all hits that have no mismatches, but that contain Wobble base-pairings. The last table contains hits with both mismatches and Wobble base pairs. The information displayed in each table includes the cDNA ID, the cDNA's name, the recognition sequence match and other information specific for each RNA tool. The ID for each hit is a link to a more detailed view of the potential substrate. This view presents essential information about the targeted substrates, including a description of the targeted mRNA. Depending on the interpretation of the header provided with the cDNA database, this may also include a link to the external source information (e.g. the NCBI detailed view of the mRNA). Other information displayed shows the position within the mRNA of the sequence targeted by the designed ribozyme. This position is mapped in color on the sequence. This module also permits the user to store the substrates in an Excel file.

The user can either wait online in order to receive the result, or it may be delivered via an e-mail message sent by the system once the computation is completed. For relatively small cDNA databases, the search is almost instantaneous, while for larger ones it takes few minutes. New RNA tools (i.e. ribozymes, deoxyribozymes, antisense DNAs and RNAs, etc.) can easily be integrated since only two configurations files must be edited for full RNA tool integration into the software. All web pages related to this newly integrated RNA tool are automatically generated from these files.

## Results & Discussion

Since the current state of knowledge of the features involved the substrate specificities of siRNAs is limited, ribozymes represent a suitable RNA silencing tool with which to illustrate the potential of this software. More specifically, we have developed examples for both SOFA-HDV ribozymes and hammerhead ribozymes. Moreover, a signature file is also available for siRNA search.

The SOFA-HDV ribozymes (SOFA stands for Specific On/oFf Adaptor) are an improved generation of ribozymes that possess significant potential in both functional genomics and gene therapy [15,17]. These ribozymes recognize their substrates through the formation of two stems: i. the P1 stem that is composed of one GU Wobble base pair followed by six consecutive Watson-Crick base pairs; and, ii. the biosensor composed by ten to fifteen Watson-Crick base pairs (BS; see Figure 2A). The sequence located between these two domains within the substrate is referred to as "the spacer", and its optimal length has been determined to be 1 to 6 nucleotides [16]. The presence of a longer spacer sequence provides a substrate that should be the least cleaved.

As an example, we investigated the specificity of a RiboSubstrate selected SOFA-HDV ribozyme targeting the internal ribosome entry site (IRES) of the hepatitis C virus (HCV) [16]. Once a configuration file (RiboParticularitites.xml) was created, the HDV-ribozyme was automatically integrated in RiboSubstrates. This configuration file includes specific information, such as the required presence of a guanosine residue at the 3'-end adjacent to the cleavage site, so as to permit formation of the essential GU Wobble base pair. The sequences of the P1 stem and biosensor (BS) were AUGGCUU and CGGUUCCGCAGA, respectively (SOFA-HDV-Rz-HCV-135). The maximal length of the spacer sequence was fixed at 10 nucleotides. These sequences were verified to be unique to the HCV genome. The number of mismatches permitted in the BS was 3, and the spacer (i.e. the distance between the P1 and BS stems) length varied between 1 and 10 nucleotides. As this ribozyme is to be used in human cells, an updated version of the *Homo sapiens *cDNA database, including an entry for a HCV type 1b complete genome, was selected and the search performed. The RiboSubstParticularities.xml files for the score calculation had some specific indications for SOFA-HDV ribozyme. For example, a score was included for the spacer length according to biochemical data published previously [15,16]. A spacer length between 1 and 6 nucleotides has a spacer score of 0; both the lack of a spacer (i.e. the P1 and BS sequences on the substrate are contiguous) and a spacer length between 7 and 9 has a spacer score of 4. An unspecified length is automatically attributed a spacer score of 10. The substrate display is illustrated in figure 2B (lower section). The designed ribozyme has only one perfect hit, specifically the HCV genome. After this entry, the lowest score was 116 for a predicted *Homo sapiens *mRNA (LOC440096) (Figure 2B and 2C). This hit possesses one mismatch and one Wobble base pair with SOFA-HDV-Rz-HCV. Biochemical experiments have shown that the presence of one mismatch causes a significant reduction of the cleavage activity of a SOFA-HDV ribozyme [15]. Therefore, this ribozyme should be specific for targeting the HCV IRES. In addition they were many other hits, with higher scores, that included several mismatches as well as Wobble base pairs. When the RiboSubstrates experiment was repeated using the original human database (i.e. without adding the HCV sequence) no perfect hit was retrieved. More importantly, several experiments have been performed both *in vitro *as well as in cultured cells (unpublished data, F Brière and JP Perreault). In each case SOFA-HDV-Rz-HCV-135 exhibited efficient cleavage of the HCV IRES and all data indicated that this ribozyme has a specific action. More importantly, RiboSubstrates had suggested that this SOFA-HDV ribozyme should be specific as was observed experimentally. In so doing RiboSubstrates prevented the lose of time designing other HCV specific ribozymes that also cleave human mRNAs.

A second example was developed for the hammerhead ribozyme since to date it has been the catalytic RNA motif receiving the greatest amount of attention in terms of the development of gene-inactivation systems [18]. This ribozyme possesses a substrate recognition domain that includes two stems located on either side of the cleavage site (i.e. stems I and III; Figure 3A). The sequence of stem I (i.e. the 5'end of the ribozyme) has no specific requirement. Conversely, the sequence of stem III, which corresponds to the 3' end of the ribozyme, starts with a required adenosine that base-pairs with the uridine adjacent to the residue located at the cleavage site. The latter residue can be adenosine, uridine or cytosine (symbolized by an H). Specific RiboParticularities.xml files describing the hammerhead signature to be searched for, and the substrate display, were appended to a previously written configuration file. An example of the search was performed for a hammerhead ribozyme targeting the human apoptosis-associated B-cell CLL/lymphoma 2 (Bcl2) mRNA (Figure 3B and 3C; [19]). The results display shows that, in addition to targeting the Bcl2 mRNA with a perfect score, this ribozyme would also have the potential to target both another variant of Bcl2 (score of 10) and the G protein-coupled receptor 97 (score of 50). Such a result provides the information required for the research decide whether or not to go further with this ribozyme, or to design another ribozyme that would be more specific for the Bcl2 mRNA. At a minimum, this result should raise a red flag, suggesting to the researcher that he should consider other information, such as if it is known that alternative targets are also expressed in the cell cultures used, before proceeding.

Finally, a template was developed for the siRNA search. Since the current knowledge does not provide an unambiguous description of the features that define the rules for siRNA specificity, only one parameter was considered. The minimal size of the siRNA should be at least 19 nucleotides. As rules become available, this signature file will be upgraded, which explains the indication "in development" appearing below the siRNA template available in the software.

## Conclusion

The avoidance of any off-target effect (i.e. non-specific cleavage) is crucial to the development of a useful RNA-based tool for gene inactivation. Given this fact, RiboSubstrates is an essential tool for the rapid selection of sequences suitable as targets for RNA degradation. This flexible and reliable program is easily adaptable for any RNA's tool specifications, and may use information from any cDNA bank. In addition the same framework could be used for selecting targets for other ribozymes, deoxyribozymes and antisense-based RNA tools. Requests for the additional development of RiboParticularities.xml files for other RNA tools, and the addition of cDNA databases, will be performed primarily in collaboration with users and the authors (please contact Jean-François Lucier or Jean-Pierre Perreault). Alternatively, we are willing to share the code to all interested scientists.

## Availability and requirements

The RiboSubstrates resource is platform that is independent and freely available for all non-commercial users at  The software has been programmed in PERL and is licensed under GNU LGPL. This web site is the property of the Sherbrooke Ribo-Club group and includes many other interesting tools and information, as well as a forum dedicated to RNA scientists. The cDNA databases will be updated frequently as new versions become available.

## List of Abbreviations

BCL2 Human apoptosis-associated B-cell CLL/lymphoma 2

HCV Hepatitis C Virus

HDV Hepatitis D Virus

SOFA Specific On/oFf Adaptor

## Authors' contributions

JFL designed the software, participated in all phases of research and contributed to write the manuscript; LJB and FPB contributed to software design and testing; RO and SAB provided intellectual contributions and revised the manuscript; JPP initiated the idea, provided intellectual contributions, supervised all aspects of the work and wrote the manuscript. All authors read and approved the final manuscript.
